# Alginate Oligosaccharide: A Promising Functional Additive for Growth, Intestine Function, Immunity, Antioxidation and Apoptosis Modulation in Largemouth Bass (*Micropterus salmoides*)

**DOI:** 10.3390/antiox15091059

**Published:** 2026-08-25

**Authors:** Hualiang Liang, Lu Zhang, Yuqun Li, Dongyu Huang, Qunlan Zhou, Xiaodu Xu, Mingchun Ren, Xiaoru Chen

**Affiliations:** 1 Key Laboratory of Integrated Rice-Fish Farming Ecology, Ministry of Agriculture and Rural Affairs, Fresh-Water Fisheries Research Center, Chinese Academy of Fishery Sciences, Wuxi 214081, China; 2 Tongwei Agricultural Development Co., Ltd., Key Laboratory of Aquatic Nutrition and Smart Farming, Ministry of Agriculture and Rural Affairs, Aquatic Health and Intelligent Aquaculture Key Laboratory of Sichuan Province, Chengdu 610093, China; 3 Wuxi Fisheries College, Nanjing Agricultural University, Wuxi 214081, China

**Keywords:** alginate oligosaccharides, juvenile largemouth bass, intestine function, antioxidant ability, immunity ability

## Abstract

A 56-day feeding trial was designed to investigate the effects of alginate oligosaccharide (AOS) on the growth, immune response, antioxidant activity and apoptosis pathways of largemouth bass (*Micropterus salmoides*). We formulated six isonitrogenous and isoenergetic diets with different concentrations of AOS (0% (control), 0.05%, 0.1%, 0.15%, 0.2% and 0.25%). The results showed that the WGR of the AOS0.15–0.2 groups were markedly increased, and the FBW and SGR of the AOS0.1–0.2 groups were also markedly boosted. In addition, no significant differences were observed in FCR, SR and FI in the treatment groups. According to SGR and WG second-degree polynomial regression analysis, the optimum AOS addition level for juvenile largemouth bass was 0.14–0.15%. On the other hand, no notable differences were observed in crude protein, moisture, crude lipid or crude ash content between groups, and no notable differences were also observed in the levels of AST and ALT in the plasma between all groups. Additionally, ALP activities were considerably higher in the AOS0.15–0.25 groups. In terms of intestinal digestion and absorption function, AOS0.1 group and AOS0.15 group significantly increased the intestinal amylase and lipase activities, and A0S0.1–0.2 groups significantly increased the intestinal trypsin activities, while proper dietary supplementation with AOS significantly improved villus muscular thickness, villus height, and villus width. Furthermore, proper dietary supplementation with AOS significantly up-regulated the mRNA levels of *occ*, *clau*, *C6A6*, *C7A5*, *C7A8B*, *C6A14* and *pept1* in the intestine. No significant differences were observed in the mRNA levels of C7A6, C7A1A and C7A10A between all groups. With respect to the antioxidant and immune functions of the intestine, the analysis revealed no remarkable differences between the groups concerning SOD, GPX activity or T-AOC content in the intestine. However, a significant increase in CAT activity of the intestine was observed in the AOS0.05–0.15 groups, and MDA levels were lower in all AOS-added groups. Apart from the above, AOS0.15–0.25 groups significantly reduced intestinal TNF-α concentration. No notable differences were observed in the intestinal contents of TGF-β, IL-10 and IL-6 between all groups. Additionally, proper dietary supplementation with AOS could improve antioxidant effects and inhibit inflammation by regulating the gene expressions of the related-Nrf2 and NF-κB signaling pathway, including *nrf2*, *keap1*, *Mn-sod*, *gpx*, *nf-κb*, *il-10* and *tgf-β*. There was no significant difference in the mRNA levels of *cat*, *fox*, *il-8* and *tnf-α.* With respect to cell apoptosis in the intestine, TUNEL assay results showed that green positive cells were significantly lower in the AOS0.05–0.2 groups than the AOS0 group. Additionally, proper dietary supplementation with AOS could inhibit cell apoptosis by regulating the mRNA levels of *bxl-xl*, *caspase 3*, *caspase 8*, *caspase 9* and *bcl-2*. However, there was no significant effect on the level of *bax* mRNA in any of the treatment groups. In summary, proper dietary supplementation with AOS exerted positive effects on growth, intestinal digestion and absorption function, immune antioxidant responses, and apoptosis pathways to a certain extent.

## 1. Introduction

The largemouth bass (*Micropterus salmoides*), a common freshwater fish farmed for food, was introduced to China from North America in 1983. It is favored by farmers and consumers due to its tender flesh, nutritional value, and rapid growth [1]. It has emerged as one of China’s most important freshwater aquaculture species. According to the China Fisheries Yearbook 2025 [2], the annual national production of largemouth bass reached 938,500 tons in 2024, an increase of 5.68% compared to 2023. Along with the increasing demand for this species, the challenges faced by farmers are to obtain an increase in growth rate. However, as intensive aquaculture expands, aquaculture density may cause environmental pollution, compromising their immune defenses against various diseases [3] and greatly increasing the likelihood of diseases spreading. In addition, the use of dietary supplements such as probiotics, Chinese herbs, and prebiotics could improve the production performance and overall health of aquaculture species [4,5]. These supplements also contribute to enhanced feed efficiency, reduced environmental impact from aquaculture activities, and increased environmental and economic sustainability [6]. Hence, it is crucial to find new biologically active substances that could replace antibiotics, particularly in aquaculture [7,8,9].

Prebiotics can be environmentally and consumer-friendly alternatives to confront this critical situation. Research has shown that adding prebiotics to feed can boost the immune systems of aquatic animals and encourage growth [10,11,12]. Oligosaccharides can be fermented by beneficial microorganisms in fish to produce short-chain fatty acids, which improve intestinal health, promote growth, and regulate immune function [8,10]. They are an important prebiotic that is widely used in aquaculture. Alginate oligosaccharides (AOSs) are functional oligosaccharides that are produced when alginate is broken down. As a new-generation prebiotic, AOS has properties such as a lower molecular weight and higher solubility [10]. In addition, AOS possess a variety of biological properties, including antioxidant [13,14], anti-apoptotic [15], anti-inflammatory [16], and antimicrobial properties [17,18].

In the last few years, AOS has become increasingly popular in aquaculture, and the beneficial effects of AOS on fish have been documented in several studies. It has previously been demonstrated that dietary supplementation with AOS enhances the growth performance of various aquatic species, including grass carp (*Ctenopharyngodon idella*) [19], Nile tilapia (*Oreochromis niloticus*) [20], Pacific white shrimp (*Litopenaeus vannamei*) [21], and thin-lip gray mullet (*Liza ramada*) fingerlings [22]. Beyond growth enhancement, AOS has been demonstrated to bolster innate immunity, as evidenced by its anti-inflammatory effects and improved survival rates in grass carp [19], white shrimp [21], thin-lip gray mullet fingerlings [22] and Chinese sea bass (*Lateolabrax maculatus*) [23]. Moreover, AOS has been shown to modulate oxidative stress responses in spotted sea bass, juvenile golden pompano (*Trachinotus ovatus*), and grass carp by enhancing antioxidant enzyme activities and regulating the Nrf2-Keap1 signaling pathway [23,24,25]. Additional investigations highlight its role in intestinal health when used in appropriate doses, such as promoting intestinal development in thin-lip gray mullet fingerlings [22], European sea bass (*Dicentrarchus labrax*) [26], and juvenile Asian sea bass (*Lates calcarifer*) [27], as well as modulating gut microbiota composition in Atlantic salmon (*Salmo salar*) [28]. Despite these documented benefits of aquatic species, no systematic study has yet investigated the impact of AOS on juvenile largemouth bass. Thus, the potential physiological impacts of AOS on this species remain unexplored and merit further investigation.

Hence, this study aimed to investigate the effects of AOS on growth, intestine function, immunity, anti-oxidation and apoptosis modulation in largemouth bass. The results will provide a new strategy for applying AOS in the largemouth bass industry and for developing green additives and aquaculture models for largemouth bass. They will also provide a theoretical basis for applying AOS to other aquaculture animals.

## 2. Materials and Methods

### 2.1. Feed Preparation

The addition levels of AOS were selected according to a previous study [21,23]. The AOS was added to diets in six different concentrations: 0% (control), 0.05%, 0.1%, 0.15%, 0.2% and 0.25%. These diets were referred to as AOS0, AOS0.05, AOS0.1, AOS0.15, AOS0.2, and AOS0.25. Table 1 lists the feed composition. There were several ingredients that acted as protein sources, with fish meal being the major one (Table 1). The primary lipid source was fish oil. All ingredients except fish oil were ground to pass through a screen size of 0.18 mm, then the mixture was blended thoroughly with fish oil at the designed levels, and pelleted into an appropriate size (2 mm) through a lab pelletizer (F-26 (II), South China University of Technology, Guangzhou, China) to produce pellet feed. After drying, the feed was kept frozen at −20 °C until needed.

### 2.2. Experimental Procedure

Research was performed at the Wuxi Fishery College, Nanjing Agricultural University (Wuxi, China). The experimental fish were acclimatized to the culture environment and fed for 14 days on a commercial diet (50% protein) provided by the Wuxi Tongwei Feedstuffs Co., Ltd. (Wuxi, China). After this period, healthy fish (body weight 9.37 ± 0.06 g) were selected and randomly assigned to 18 cages (1 m × 1 m × 1 m), each containing 20 fish. Apparent satiation feeding (stop coming up to feed) was achieved by manually administering feed at 07:30 and 17:30 every day for 56 days. Daily records were kept of the survival rate and the weight of any fish that died in each cage. Water quality parameters were measured weekly using a YSI ProPlus multiparameter water quality analyzer (YSI China, Hong Kong, China). The experiment was conducted with water temperature at 29.0 ± 1.0 °C, the dissolved oxygen at ≥6 mg/L, the pH at 7.0–7.8, and ammonia nitrogen and nitrite concentrations ≤ 0.02 mg/L and ≤0.3 mg/L.

### 2.3. Sample Collection

After the eight-week feeding trial, all the experimental fish were subjected to a 24 h fasting period. The total weight of the fish in each cage as the experimental one unit for growth-related outcomes was then recorded in order to calculate growth parameters. Three fish were randomly sampled from each cage and anesthetized with 100 mg/L MS-222. After loss of equilibrium, the blood and intestine of fish were rapidly collected. First, blood was drawn from the caudal vein and immediately centrifuged for 10 min (1700× *g*, 4 °C). Then, upper plasma samples were obtained and stored in a −20 °C freezer. A total of nine plasma samples per group were used for plasma biochemical analysis. Intestinal samples from three fish per cage were collected by dissection, respectively. A portion of nine intestinal samples per group was stored in 4% paraformaldehyde for pathological analysis, respectively. The remaining nine intestinal samples per group were stored in a −80 °C freezer, which were used for gene and enzymatic activity analysis, respectively. Two fish from each cage were stored at −20 °C for subsequent whole-body composition analysis, respectively.

### 2.4. Laboratory Analysis

The moisture, crude protein, crude lipid, and ash contents (%) of the experimental feed and whole fish body were measured using standard methods [29]. Plasma ALT, ALP, and AST levels were analyzed using a Mindray BS-400 automatic biochemical analyzer (Shenzhen, China). Accurately weigh intestinal tissue samples, and add 9 volumes of 0.9% normal saline at a weight (g): volume (mL) ratio of 1:9. After mechanical homogenization, the homogenate was centrifuged, and the supernatant was collected to prepare 10% tissue homogenate for subsequent analysis. Prior to the detection of antioxidant-related indicators, the total protein (TP) content in intestinal tissue was determined via the Bradford method [30]. The detection principle is as follows: the reddish-brown coomassie brilliant blue chromogenic reagent binds to the -NH_3_^+^ groups on protein molecules in tissue homogenate, turning the solution blue. The protein concentration is calculated by measuring absorbance, while intestinal antioxidant parameters (SOD, T-AOC, CAT, GPX, and MDA) and intestinal digestive enzyme levels (amylase, lipase and trypsin) were measured using assay kits from the Nanjing Jiancheng Bioengineering Institute (Nanjing, China) [31], and the detailed methods are shown in Table 2. First, the whole fish body samples and feed were dried in an oven at 105 °C to determine their moisture content. Crude protein content was then measured using the Kjeldahl method on an automatic analyzer (Haineng K1100, Jinan Haineng Instrument Co., Ltd., Jinan, China). The crude fat content was determined using the Soxhlet extraction method and an automatic fat analyzer (Haineng SOX606, Jinan Haineng Instrument Co., Ltd., Jinan, China). Additionally, the samples were ashed at 560 °C for 6 h in a muffle furnace (XL-2A, Hangzhou Zhuochi Instrument Co., Ltd., Hangzhou, China) to obtain the ash content. Enzyme-linked immunosorbent assays were adopted to detect inflammatory factors in intestine [32]. The detection was performed following the manufacturer’s protocols. Table 2 provides a summary of the main kits, key experimental apparatus, and primary methodological procedures used.

### 2.5. Histomorphological Analysis and Enterocyte Apoptosis Detection

We removed the fixed intestinal tissues from 4% formaldehyde, processed them according to standard histological techniques, dehydration, waxing, burying, and slicing; the samples were then subjected to staining with hematoxylin–eosin (H&E). Two slices were made for each processing group, and the intestinal tract was observed and photographed using a Nikon biomicrographic system (Tokyo, Japan). The intestinal villus height, villus width and muscular thickness of the intestine were measured using Image-Pro Plus 6.3 software (Media Cybernetics, Inc., Rockville, MD, USA).

The fixed intestinal tissues were removed from 4% paraformaldehyde and washed with PBS before being permeabilized. After pretreatment, the samples were incubated with the TUNEL reaction mix. The samples were prepared according to the recommended ratio in the kit, with the addition of TdT enzyme and fluorescently labeled dUTP (usually FITC or rhodamine). After incubation, the samples were washed three times with PBS for five minutes each time to remove unbound dUTP and TdTase. After washing, the samples were allowed to dry under light-protected conditions and then sealed with a fluorescence quenching-resistant sealer. Use a fluorescence microscope to observe the samples and obtain images. Apoptotic cells will appear as positive fluorescent signals. Quantify the fluorescent signal using image analysis software such as ImageJ (Version 1.54r). Calculate the apoptotic cell index by dividing the number of fluorescence-positive cells by the total number of cells.

### 2.6. Isolation of Total RNA and Quantitative Real-Time RT–PCR Analysis

Total RNA was isolated from the intestinal tissue of largemouth bass using the RNAiso Plus kit (Vazyme, Nanjing, China). The concentration and quality of the total RNA were then assessed using a spectrophotometer (NanoDrop 2000, Thermo Fisher Scientific, Inc., Waltham, MA, USA). High-quality total RNA has an absorbance ratio of 260/280 nm between 1.8 and 2.0. Each RNA sample was subsequently normalized to a uniform concentration and a standard curve was generated using serial two-fold dilutions for quantitative reference. The primer sequences are provided in Table 3. All qPCR primers in this study were fully validated. The specificity of each primer pair was confirmed via melting curve analysis, which revealed no non-specific amplification or primer-dimer formation. Standard curves were constructed using seven 2-fold serial dilutions of RNA template to calculate amplification efficiency and R^2^ values. The amplification efficiencies of all primer pairs ranged from 95.6% to 109.2%, with R^2^ > 0.99, satisfying the quantitative criteria for qPCR. All relevant parameters have been summarized in Table 3. The qPCR was performed using HiScript^®^ II One Step qRT-PCR SYBR Green Kit (Q221-01, Vazyme, Nanjing, China) on a CFX96 Touch (Bio-Rad, Hercules, CA, USA). The qPCR reactions were programmed as follows: 50 °C for 15 min, 95 °C for 30 s, and 40 cycles were performed at 95 °C for 10 s and 60 °C for 30 s, then after the cycles were finished, 95 °C for 15 s, 60 °C for 60 s, and 95 °C for 15 s. Based on its consistent expression across experimental conditions, *gapdh* was selected as the internal reference gene. Target gene abundance was determined using the relative standard curve approach. Standard curves were constructed from serial dilutions of a reference template and sample Ct values were then interpolated against these curves.

### 2.7. Statistical Analysis

Shapiro–Wilk and Levene tests were used before statistical analysis to evaluate the normality and homogeneity of variance of the data, respectively, and the findings satisfied the criteria for further analysis. A one-way analysis (ANOVA) was performed on the data using the SPSS software (version 20.0), and the comparison of differences between groups was performed using Tukey’s test. Results are shown as mean ± standard deviations (SD), with figures produced using GraphPad Prism 9.5.1 (GraphPad, La Jolla, CA, USA). Statistical significance was set at *p* < 0.05.

## 3. Results

### 3.1. Growth Performance

**Figure 1:**
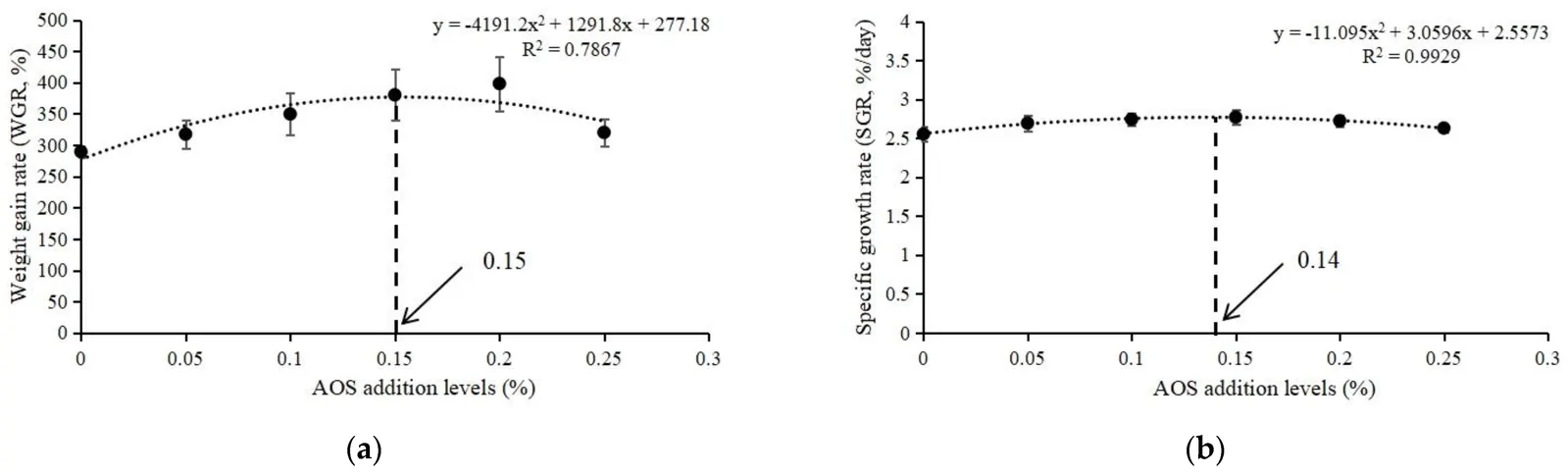
Second-degree polynomial regression analysis of weight gain rate (WGR, %) and specific growth rate (SGR, %/day) for juvenile largemouth bass against AOS addition level. (**a**) weight gain rate (WGR, %); (**b**) specific growth rate (SGR, %/day).

Table 4 shows the growth performance. The results show that, compared with the AOS0 group, the weight gain rate (WGR) of the AOS0.15–0.2 groups were markedly increased, and the final body weight (FBW) and specific growth rate (SGR) of the AOS0.1–0.2 groups were also markedly boosted (*p* < 0.05). In addition, no significant differences were observed in feed conversion ratio (FCR), survival rate (SR), and feed intake (FI) in the treatment groups (*p* > 0.05). According to SGR and WG second-degree polynomial regression analysis, the optimum AOS addition level for juvenile largemouth bass was 0.14–0.15% (Figure 1).

### 3.2. Whole-Body Composition

Table 5 demonstrates the whole-body composition. No notable differences were observed in crude protein, moisture, crude lipid or crude ash content between groups (*p* > 0.05).

### 3.3. Plasma Parameters

**Figure 2:**
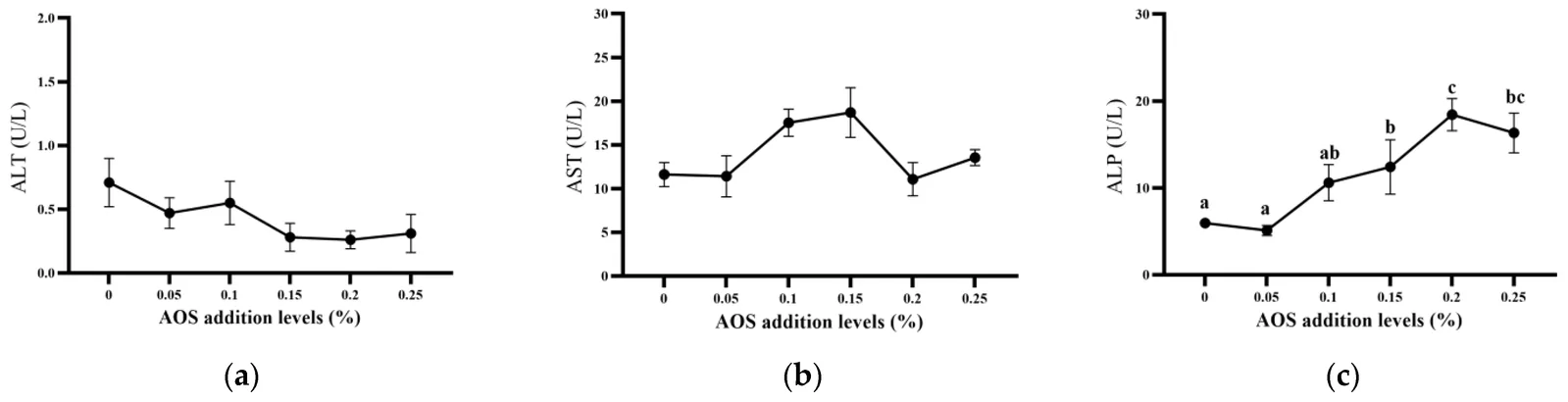
Plasma parameters of juvenile largemouth bass fed on alginate oligosaccharide (AOS)-supplemented diets for 56 days. Data are mean value ± SD, different letters indicate significant differences (*p* < 0.05). (**a**) ALT (U/L); (**b**) AST (U/L); (**c**) ALP (U/L).

The plasma parameters are displayed in Figure 2. There were comparable activities of AST and ALT across the treatment and control groups, with no notable differences (*p* > 0.05). Additionally, ALP activities were considerably higher in the AOS0.15–0.25 groups (*p* < 0.05).

### 3.4. The Levels of Intestinal Digestive Enzymes, Antioxidant Indexes, and Inflammatory Markers

#### 3.4.1. The Levels of Intestinal Digestive Enzymes

**Figure 3:**
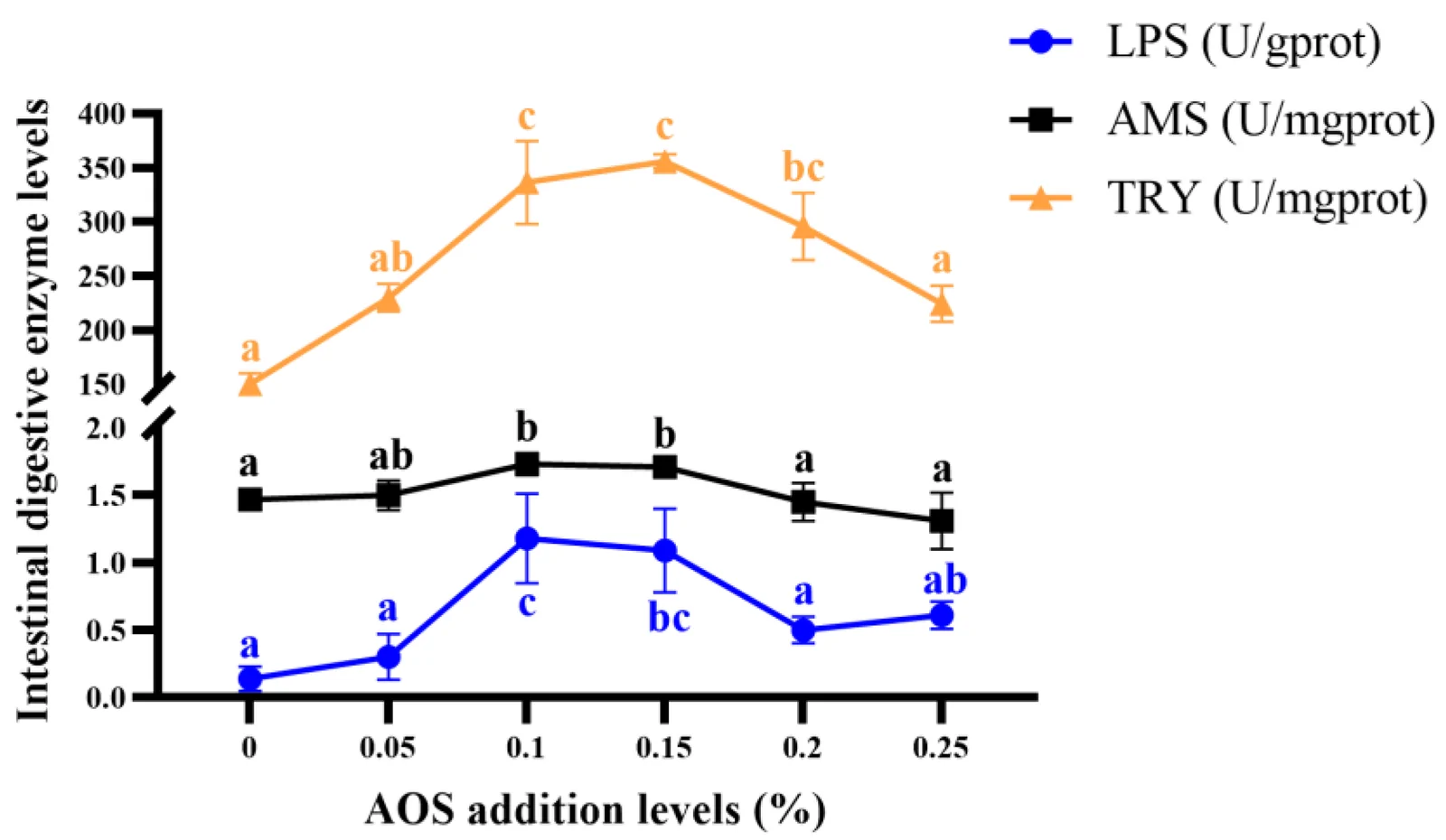
Intestinal digestive enzyme of juvenile largemouth bass fed on alginate oligosaccharide (AOS)-supplemented diets for 56 days. Data are mean value ± SD, different letters indicate significant differences (*p* < 0.05).

It can be seen that the activities of AMS, LPS and TRY were significantly affected by dietary AOS levels, showing a trend of first increasing and then decreasing. Compared with the AOS0 group, the activities of AMS and LPS were significantly increased in the AOS0.1 group and AOS0.15 group (*p* < 0.05), and the TRY activity was markedly elevated in the AOS0.1–0.2 groups (*p* < 0.05) (Figure 3).

#### 3.4.2. The Levels of Intestinal Antioxidant Indexes

**Figure 4:**
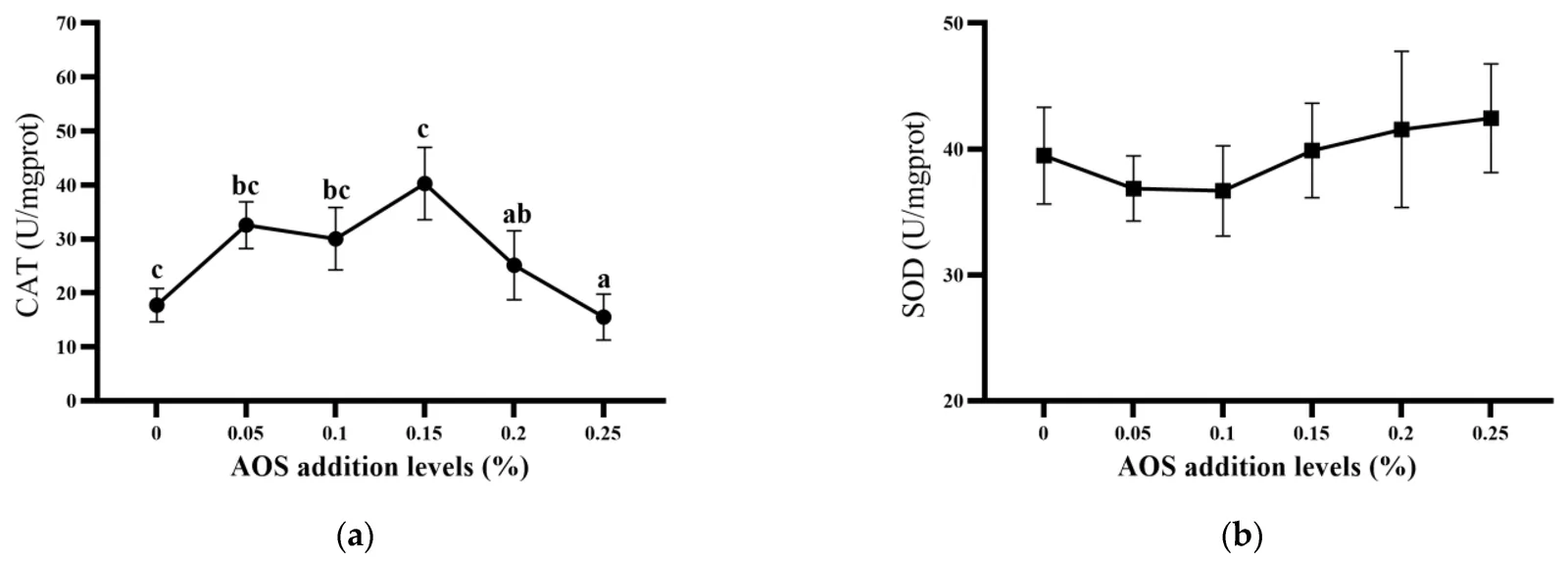

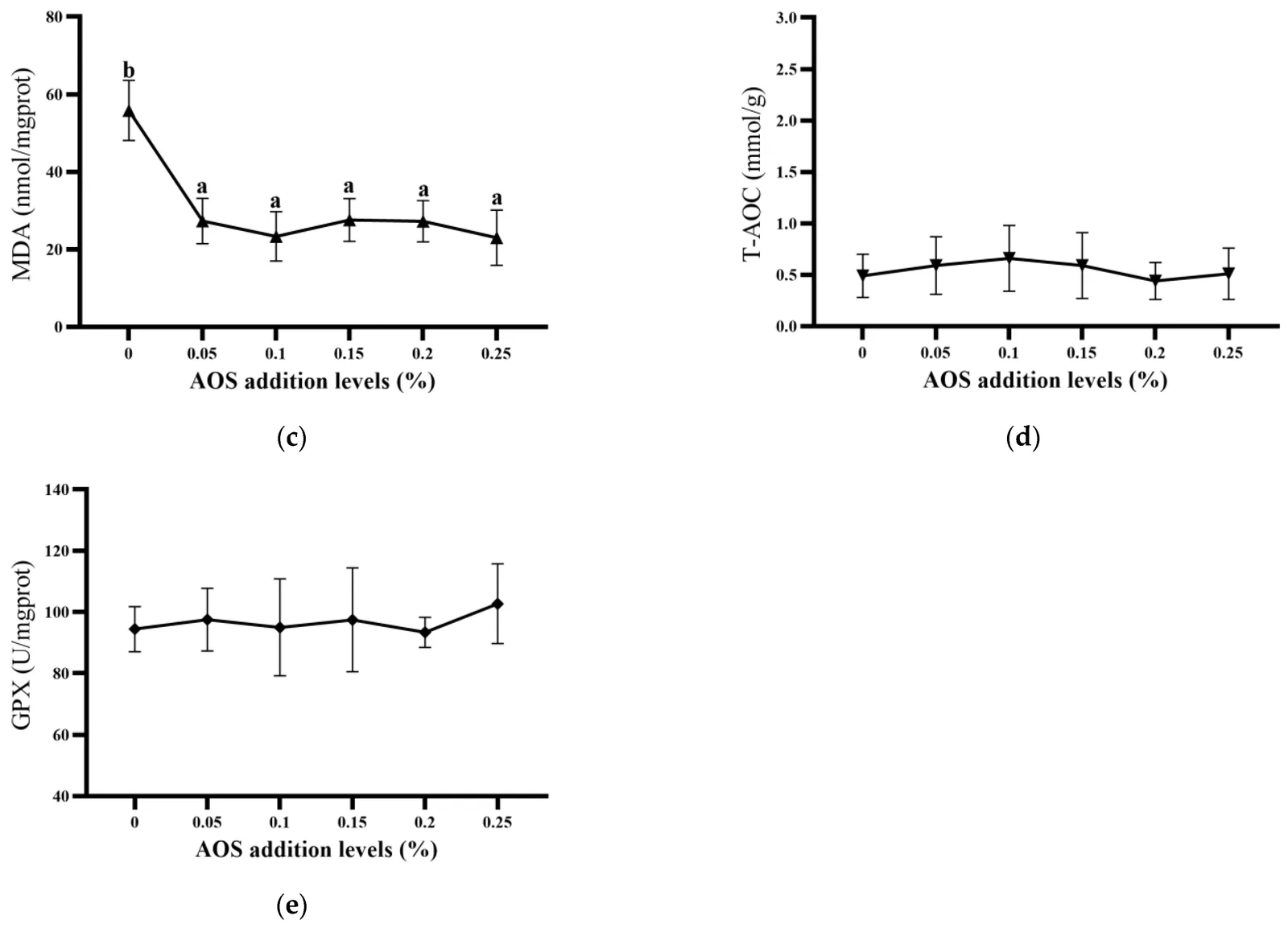
Intestinal antioxidant indexes of juvenile largemouth bass fed on alginate oligosaccharide (AOS)-supplemented diets for 56 days. Data are mean value ± SD, different letters indicate significant differences (*p* < 0.05). (**a**) CAT (U/mgprot); (**b**) SOD (U/mgprot); (**c**) MDA (nmol/mgprot); (**d**) T-AOC (mmol/g); (**e**) GPX (U/mgprot).

The CAT activity in the AOS0.05–0.15 groups was a lot higher than in the AOS0 group, and the difference was significant (*p* < 0.05). In addition, no notable differences were observed in the GPX, SOD activity, and T-AOC content between groups (*p* > 0.05). The MDA content was significantly lower in all treatment groups compared to the AOS0 group (*p* < 0.05) (Figure 4).

#### 3.4.3. The Levels of Intestinal Inflammatory Markers

**Figure 5:**
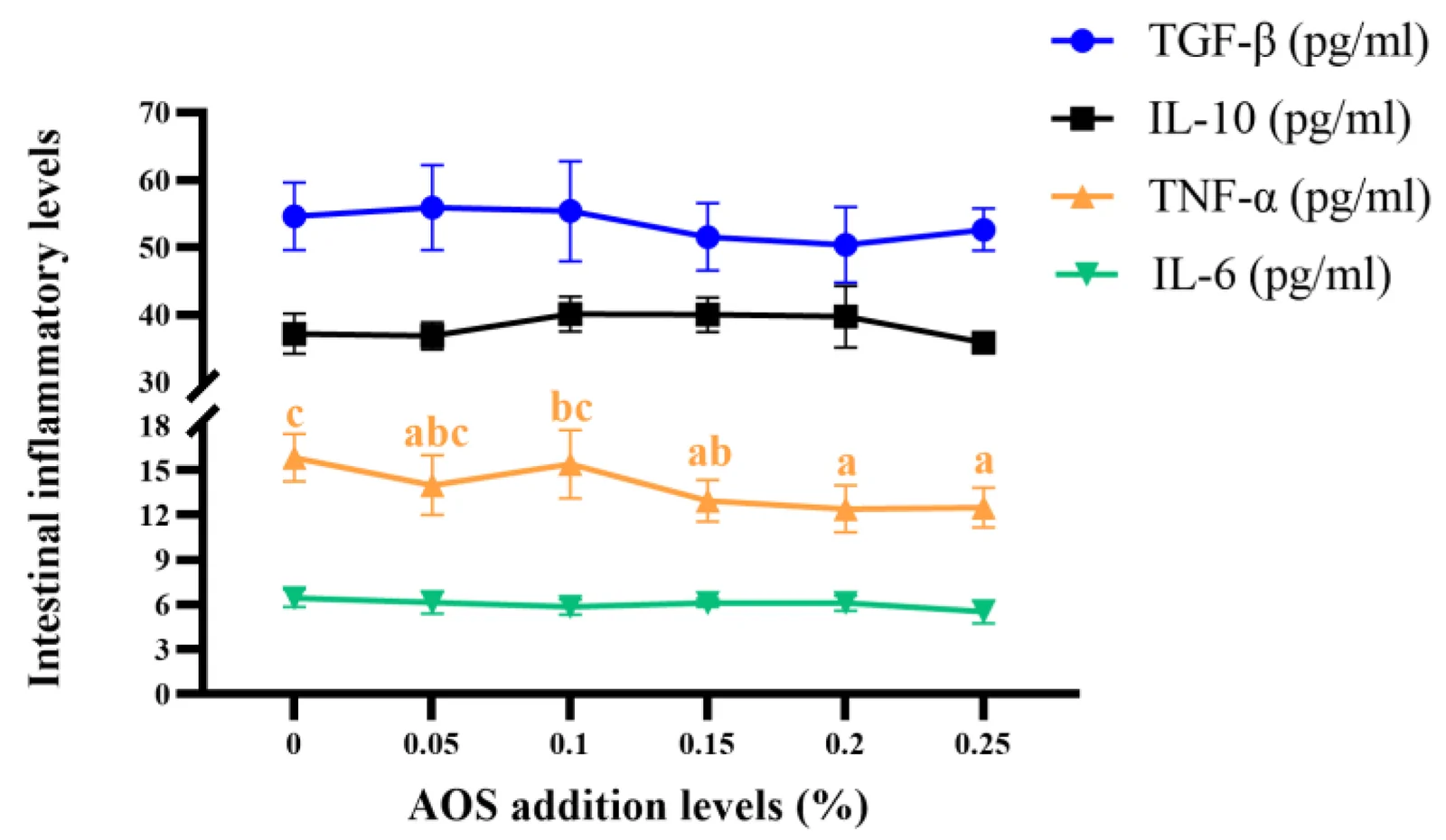
Intestinal inflammatory markers of juvenile largemouth bass fed on alginate oligosaccharide (AOS)-supplemented diets for 56 days. Data are mean value ± SD, different letters indicate significant differences (*p* < 0.05).

No notable differences were observed in the intestinal contents of TGF-β, IL-10 and IL-6 between all groups (*p* > 0.05). Compared with the AOS0 group, the TNF-α content was significantly decreased in the AOS0.15–0.25 groups (*p* < 0.05) (Figure 5).

### 3.5. The mRNA Levels of Gene-Related Intestinal Tight Junction Proteins and Transporters, and Antioxidant and Inflammatory Capacity

#### 3.5.1. The mRNA Levels of Intestinal Tight Junction Proteins and Transporters

**Figure 6:**
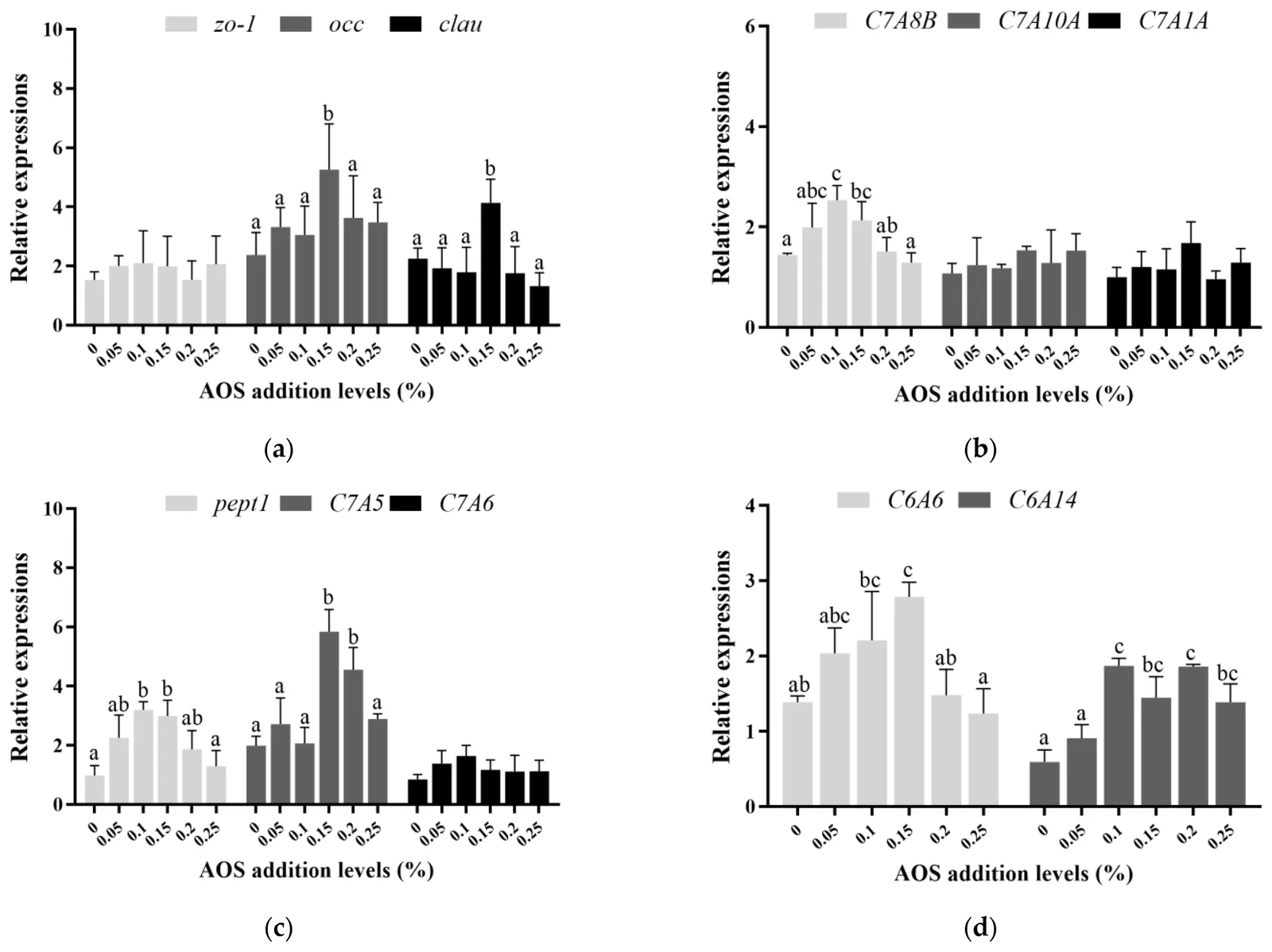
Intestinal tight junction protein- and transporter-related gene expressions of juvenile largemouth bass fed on alginate oligosaccharide (AOS)-supplemented diets for 56 days. Data are mean value ± SD, different letters indicate significant differences (*p* < 0.05). (**a**) *zo-1*, *occ* and *clau*; (**b**) *C7A8B*, *C7A10A* and *C7A1A*; (**c**) *pept1*, *C7A5* and *C7A6*; (**d**) *C6A6* and *C6A14*.

Figure 6 displays the mRNA levels of intestinal tight junction proteins and transporters. The mRNA levels of *occ* and *clau* were considerably higher in the AOS0.15 group (*p* < 0.05), though there was no difference in *zo-1* mRNA levels (*p* > 0.05). In terms of transporter, compared with the AOS0 group, the mRNA levels of *pept1*, *C6A6* and *C7A8B* were significantly up-regulated in the AOS0.1–0.15 groups (*p* < 0.05). In addition, the mRNA levels of *C7A5* in the AOS0.15–0.2 groups was markedly higher than that in the AOS0 group, and the mRNA levels of *C6A14* in the AOS0.1–0.25 groups was also significantly elevated relative to the AOS0 group (*p* < 0.05). No significant differences were observed in the mRNA levels of *C7A6*, *C7A1A* and *C7A10A* between all groups (*p* > 0.05).

#### 3.5.2. The mRNA Levels of Intestinal Antioxidant and Inflammatory Capacities

**Figure 7:**
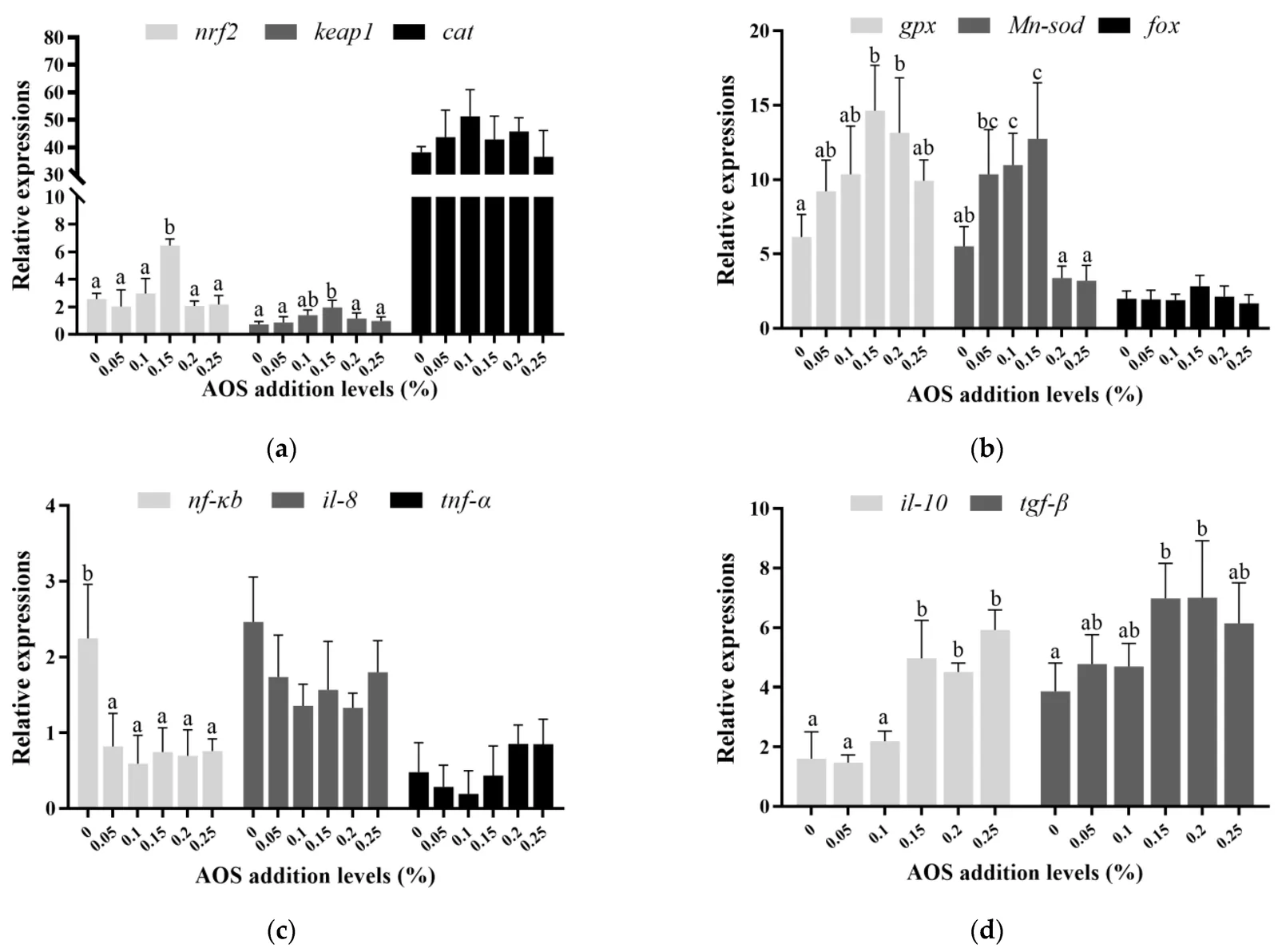
Intestinal antioxidant- and inflammatory-related gene expressions of juvenile largemouth bass fed on alginate oligosaccharide (AOS)-supplemented diets for 56 days. Data are mean value ± SD, different letters indicate significant differences (*p* < 0.05). (**a**) *nrf2*, *keap1* and *act*; (**b**) *gpx*, *Mn-sod* and *fox*; (**c**) *nf-κb*, *il-8* and *tnf-α*; (**d**) *il-10* and *tgf-β*.

Figure 7 displays the mRNA levels of intestinal antioxidant and inflammatory capacities. In terms of antioxidant capacity, the AOS0.15 group exhibited markedly higher *nrf2* and *keap1* mRNA levels (*p* < 0.05). Additionally, considerable up-regulation of *Mn-sod* mRNA levels was observed in the AOS0.05–0.15 groups (*p* < 0.05), as well as increased *gpx* mRNA levels in the AOS0.15–0.2 groups (*p* < 0.05). There was no significant difference in the mRNA levels of *cat* and *fox* (*p* > 0.05). With respect to inflammatory capacities, compared with the AOS0 group, *il-10* mRNA levels were obviously increased in the AOS0.15–0.25 groups and *tgf-β* mRNA levels were considerably higher in the AOS0.15–0.2 groups (*p* < 0.05). There was no significant difference in *il-8* and *tnf-α* mRNA levels on the addition of AOS to the feed (*p* > 0.05). Additionally, *nf-κb* mRNA levels were considerably lower in the AOS0.05–0.25 groups (*p* < 0.05).

### 3.6. The Apoptosis-Related Gene Expression and DNA Damage in Intestinal Cells

**Figure 8:**
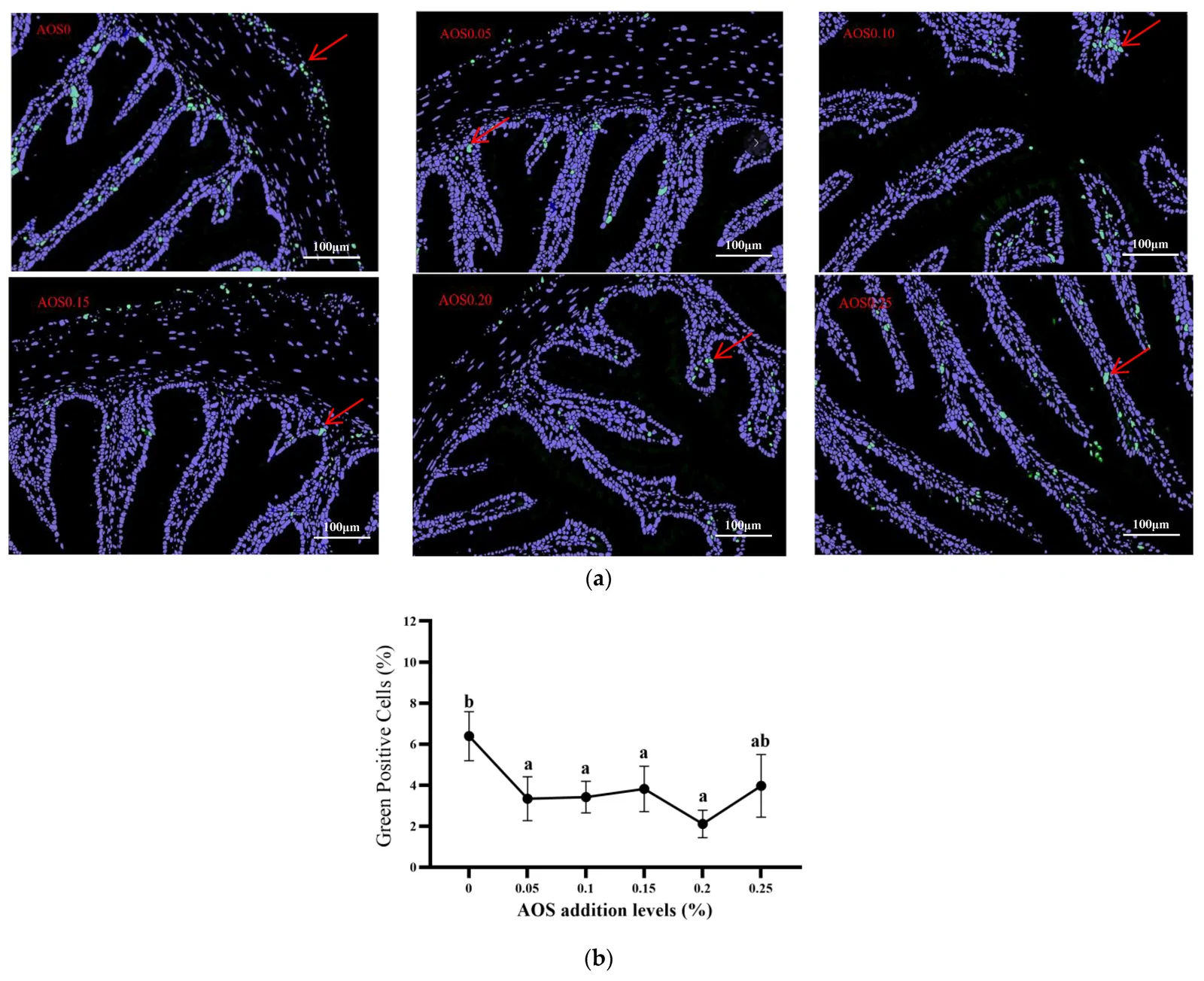
DNA damage in intestinal cells of juvenile largemouth bass fed on alginate oligosaccharide (AOS)-supplemented diets for 56 days. The red arrow points to positive cells and the scale bar is 100 µm. Data are mean value ± SD, different letters indicate significant differences (*p* < 0.05). (**a**) DNA damage in intestinal cells; (**b**) Green Positive Cells (%).

**Figure 9:**
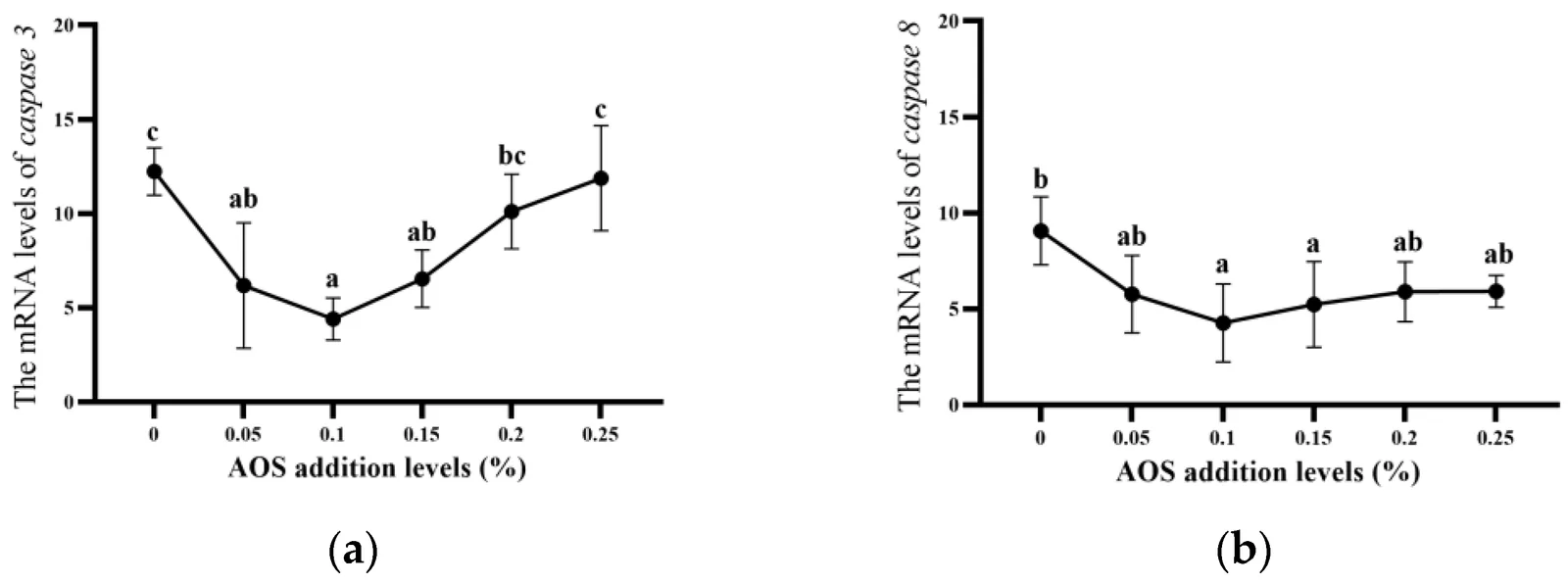

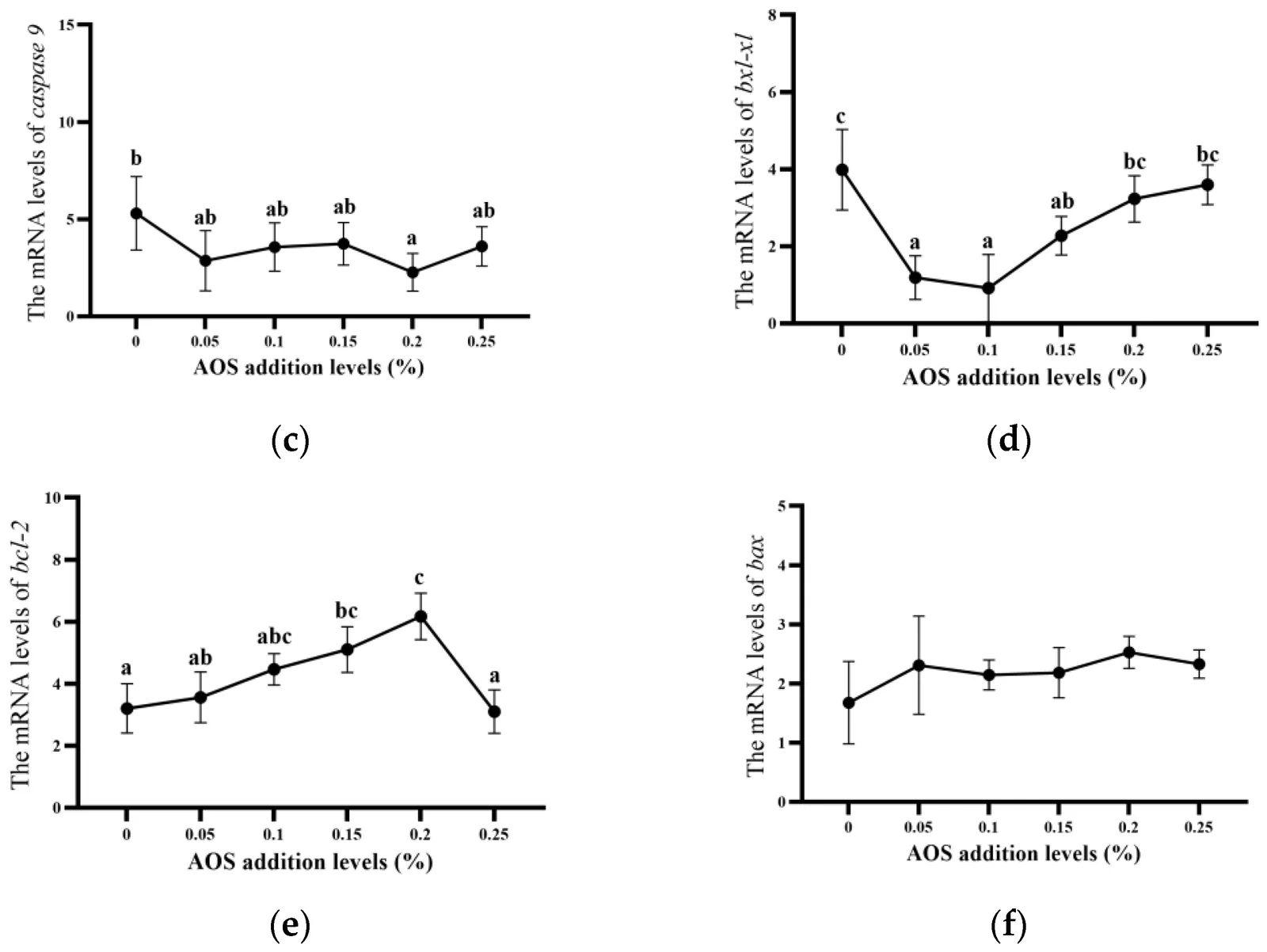
Apoptosis-related gene expressions of juvenile largemouth bass fed on alginate oligosaccharide (AOS)-supplemented diets for 56 days. Data are mean value ± SD, different letters indicate significant differences (*p* < 0.05). (**a**) *caspase 3*; (**b**) *caspase 8*; (**c**) *caspase 9*; (**d**) *bxl-xl*; (**e**) *bcl-2*; (**f**) *bax*.

Figure 8 and Figure 9 display the expression results of genes linked to apoptosis and DNA damage. Green positive cells were significantly lower in the AOS0.05–0.2 groups than the AOS0 group (*p* < 0.05). The AOS0.05–0.15 groups had considerably lower mRNA levels of *bxl-xl* and *caspase 3* in relation to apoptosis (*p* < 0.05). The mRNA levels of *caspase 8* were considerably lower in the AOS0.1–0.15 groups and mRNA levels of *caspase 9* were considerably lower in the AOS0.2 group (*p* < 0.05). Additionally, the *bcl-2* mRNA level first increased and then decreased, significantly increasing in the AOS0.15 and AOS0.2 groups (*p* < 0.05). However, there was no significant effect on the level of *bax* mRNA in any of the treatment groups (*p* >0.05).

### 3.7. Intestinal Histology

**Figure 10:**
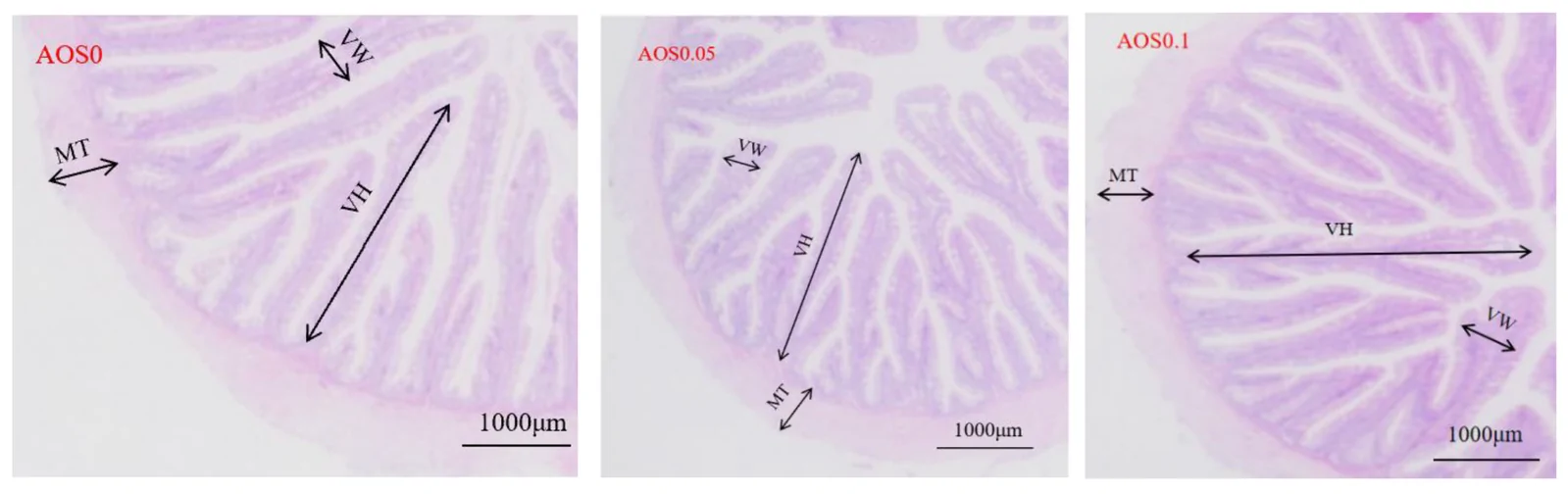

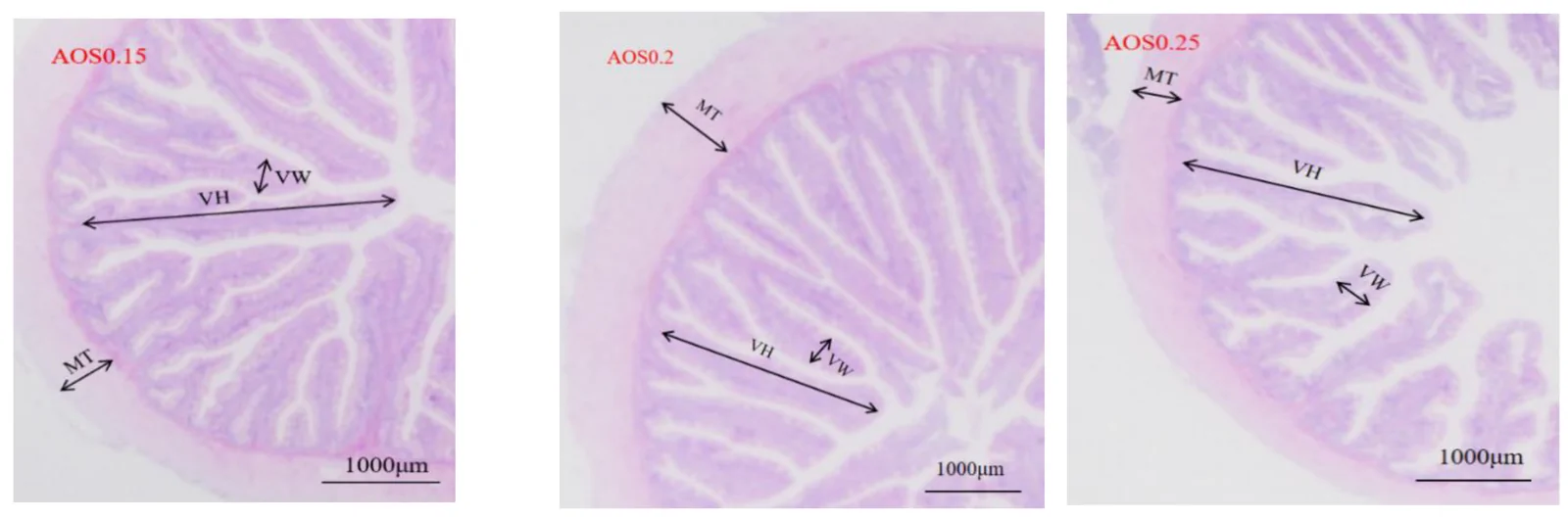
Intestinal structure of juvenile largemouth bass fed on alginate oligosaccharide (AOS)-supplemented diets for 56 days.

**Figure 11:**
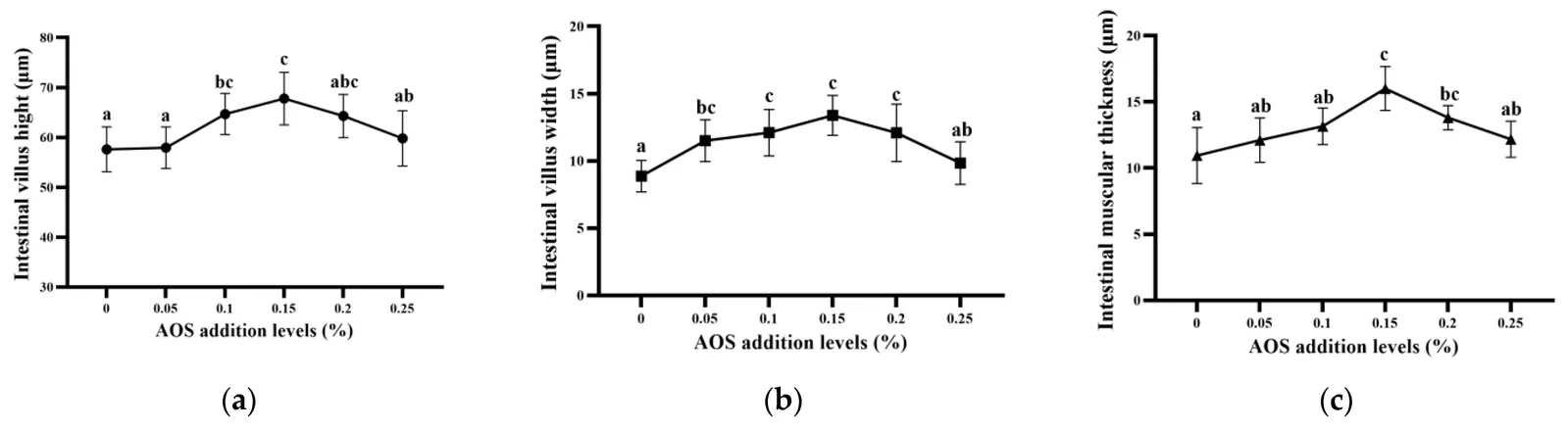
Intestinal measurements of juvenile largemouth bass fed on alginate oligosaccharide (AOS)-supplemented diets for 56 days. Data are mean value ± SD, different letters indicate significant differences (*p* < 0.05). (**a**) Intestinal villus hight (μm); (**b**) Intestinal villus width (μm); (**c**) Intestinal muscular thickness (μm).

Figure 10 and Figure 11 illustrate the intestinal histology. The results show that, compared with the AOS0 group, the villus height (VH) was significantly increased in the AOS0.1–0.15 groups, the villus width (VW) was significantly increased in the AOS0.05–0.2 groups, and the muscular thickness (MT) was markedly elevated in the AOS0.15–0.2 groups (*p* < 0.05).

## 4. Discussion

In the current study, the WGR of the AOS0.15–0.2 groups were markedly increased, and the FBW and SGR of the AOS0.1–0.2 groups were also markedly boosted. According to SGR and WG second-degree polynomial regression analysis, the optimum AOS addition level for juvenile largemouth bass was 0.14–0.15%. The favorable effects of AOS as a feed supplement have been demonstrated in a variety of fish species. Previous experiments have revealed that adding AOS to the feed promotes the growth of grass carp [19], Nile tilapia [20], thin-lip gray mullet fingerlings [22], and juvenile golden pompano [24]. Furthermore, dietary supplementation with AOS did not result in any notable changes to the overall body composition of the juvenile largemouth bass. While the impact of AOS on fish body composition has not been extensively documented, the underlying mechanisms warrant further investigation. The improved growth performance of juvenile largemouth bass following AOS supplementation was accompanied by the promotion of fish intestine function, as reflected in gut morphometry and associated enzyme activities [24,26,36]. Dietary addition of AOS was found to significantly increase the intestinal villus height of juvenile European seabass [26], Asian sea bass (*Lates calcarifer*) [27], and grouper (*Epinephelus coioides*) [37]. Additionally, dietary supplementation with appropriate oligosaccharides increased intestinal villus height and muscular layer thickness in blunt snout bream (*Megalobrama amblycephala*) [38], enhancing the fish’s digestive and absorptive capacity. Consistent with previous studies, this experiment revealed that the AOS0.1–0.15 groups significantly increased the villus length in the juvenile largemouth bass intestinal tract. The AOS0.05–0.2 groups were found to significantly increase intestinal villus width, and the AOS0.15–0.2 groups were found to significantly increase muscularis propria thickness in the intestinal tract of juvenile largemouth bass. These changes enlarged the surface area of the intestine, improving nutrient absorption capacity and further promoting growth performance [39]. Conversely, the thickening of the intestinal muscularis layer enhanced intestinal peristalsis and facilitated nutrient absorption and utilization [40,41]. Additionally, AOS appears to boost the activity of ALP, which is linked to the development of enterocytes. ALP is seen as a key marker enzyme for the major digestive and absorptive functions of the small intestine [42]. In this experiment, supplementing the feed with AOS0.15–0.25 significantly increased ALP activity. This finding is consistent with a previous study on white shrimp [21] and juvenile turbot (*Scophthalmus maximus*) [43]. This suggests that AOS enhances intestinal nutrient absorption in juvenile largemouth bass. Furthermore, we examined serum biochemical markers and found that the activities of AST and ALT remained largely unaltered in juvenile largemouth bass fed with AOS-supplement feed, which suggested that AOS does not affect the liver functions of juvenile largemouth bass.

Tight junction proteins play an important role in the physical barrier, and a decrease in these proteins has been found to increase intestinal permeability and lead to a decrease in intestinal defense [44,45,46]. In this study, the mRNA levels of *occ* and *clau* were considerably advanced in the AOS0.15 group, and the results were consistent with studies on broiler chickens [47] and rumen epithelial cells [48]. This suggests that AOS can effectively enhance the physical barriers of the largemouth bass intestinal tract, resist the invasion of pathogens and other harmful substances, and maintain normal intestinal function. Furthermore, in the present study, dietary supplementation with 0.1–0.15% AOS significantly increased the activities of LPS and AMS in the intestine of juvenile largemouth bass. Meanwhile, TRY activity was also markedly elevated in the AOS0.1–0.2 groups. These results indicate that dietary supplementation with AOS could improve the nutrient utilization efficiency of fish. Consistent with the present findings, dietary supplementation with AOS increased intestinal lipase activity in barramundi [27]. In terms of the expression of peptide transporters, dietary supplementation with AOS significantly up-regulated the mRNA levels of amino acid transporters in the intestine of juvenile largemouth bass, including *C6A6*, *C7A5*, *C7A8B*, *C6A14* and *pept1*. Collectively, dietary supplementation with AOS significantly improved the intestinal transport efficiency of small peptides and amino acids by up-regulating the expression of intestinal oligopeptide transporters and amino acid transport-related genes. This further enhanced the intestinal capacity for nutrient absorption and utilization, thereby providing sufficient nutrition to support the growth of fish. A possible mechanism is that AOS can enhance the activities of intestinal digestive enzymes and brush border enzymes. By maintaining stable pH and osmotic conditions in the intestinal tract, brush border enzymes facilitate amino acid transport, a process that relies on steady intestinal pH and the dynamic balance of sodium and potassium [49,50]. These findings reveal that AOS supplementation fosters intestinal development, enhancing digestive and metabolic capacities, as well as improving intestinal functions in juvenile largemouth bass.

Studies have shown that AOS can modulate and enhance immune function in farmed animals [51]. Fish produce a variety of inflammatory cytokines, including anti-inflammatory mediators (IL-10 and TGF-β) and pro-inflammatory factors (IL-6, IL-1β, IL-8 and TNF-α) [52]. Inflammation can be alleviated by up-regulating the levels of anti-inflammatory factors and downregulating those of pro-inflammatory factors [52]. In the present study, dietary supplementation with AOS at levels of 0.15–0.25% significantly reduced TNF-α concentration in juvenile largemouth bass; furthermore, this study also demonstrated that the addition of AOS to the feed both significantly reduced the mRNA level of *nf-κb* and significantly increased the *il-10* mRNA level in the AOS0.15–0.25 groups, while the *tgf-β* mRNA level was significantly increased in the AOS0.15–0.2 groups. However, the *il-8* and *tnf-α* mRNA level did not show a significant difference. These results indicated that dietary supplementation with AOS could improve intestinal immune function by regulating inflammatory factor levels and expressions. Previous studies have confirmed that the supplementation of feed with AOS could up-regulate *il-10* mRNA level and down-regulate *il-1β*, *il-8*, and *tnf-α* mRNA levels, thus reducing intestinal inflammation in grass carp [19]. The juvenile golden pompano fed with AOS-supplemented feed exhibited enhanced intestinal anti-inflammatory capacity by the inhibition of *il-8* and *tnf-α* mRNA level [24]. Additionally, feeding Chinese sea bass on AOS diets significantly inhibited the mRNA levels of *tnf-α*, *il-8*, and *il-1β*, and promoted the mRNA levels of *il-4* and *il-10*, thereby regulating the inflammatory response [23]. The mechanism of action may be the inactivation of NF-κB signaling in intestine, which subsequently leads to the inhibition of inflammatory mediator production [53].

In fish, the role of the antioxidant enzyme system is to eliminate excess reactive oxygen species and maintain the balance between oxidation and antioxidants, thereby protecting tissues from oxidative damage [54]. In the present study, dietary supplementation with AOS did not notably alter the activities of SOD, GPX or T-AOC. However, a notable increase in CAT activity and a significant reduction in MDA content were observed in the AOS 0.05–0.15 groups. Similar results were observed in studies on white shrimp [21], juvenile golden pompano [24] and grass carp [25]. These findings imply that the incorporation of AOS into sustenance amplifies the activity of antioxidant enzymes and the scavenging of free radicals within organisms, leading to decreased lipid peroxidation in cell membranes. It is possible that the antioxidant mechanism of AOS involves binding hydrogen from hydrogen bonds to free radicals and incorporating it into its special conjugated oleic acid structure to create a resonance-stabilized adduct that could successfully suppress free radical activity [55]. Nrf2, a crucial protein in the antioxidant defense system, is involved in regulating both the cellular stress response and antioxidant defense [55,56,57,58]. In the current experiments, a remarkable up-regulation in the mRNA levels of both *nrf2* and *keap1* were observed in the AOS 0.15 group relative to the AOS0 group. Typically, *keap1* interacts with the Neh2 domain of *nrf2*, and during oxidative stress, *nrf2* mRNA level is increased while *keap1* mRNA level is decreased. Similar findings have been observed in rainbow trout (*Oncorhynchus mykiss*) [59] and zebrafish (*Danio rerio*) [60]. However, *keap1* not only functions as a *nrf2* inhibitor, but also as one of its target genes. *nrf2*-mediated *keap1* induction represents a negative feedback mechanism, increased expression of Keap1 enhances the regulation of Nrf2 abundance, reduces its gene expression, and thereby contributes to more effective antioxidant responses [61,62,63]. Furthermore, our results showed that adding AOS to the feed raised the mRNA levels of *gpx* and *Mn-sod*. Consistent with the findings in grass carp, *sod*, and *gpx* levels were increased in grass carp fed with AOS [64], although we did not observe a significant change in the mRNA levels from *cat* and *fox*. This phenomenon may be explained by the activation of the Nrf2-Keap1 signaling pathway following AOS supplementation. It subsequently up-regulates the expression of genes that encode antioxidant enzymes. This helps to alleviate oxidative stress and support intestinal health. Overall, these findings suggest that AOS enhances the antioxidant defense system in juvenile largemouth bass by stimulating antioxidant enzyme activities and promoting the Nrf2-Keap1 signaling pathway.

Apoptosis is a form of physiological cell death that plays an important role in regulating the renewal of intestinal mucosal epithelial cells; however, if it is dysregulated or excessive, it can lead to severe intestinal lesions [65]. Apoptosis is regulated by a combination of cysteine aspartate proteases and the *bcl-2* family. *Caspase 8* or *caspase 9* can activate *caspase 3*, which is a key apoptotic protease involved in the final common pathway of the programming of cell death known as apoptosis [66]. This ultimately leads to physiological and biochemical disturbances in the cell and cell death [67,68]. The current study found that appropriate AOS increased the mRNA levels of *bcl-2* genes and exerted an anti-apoptotic effect. In addition, the mRNA level of *caspase 8* was significantly reduced in AOS0.1–0.15 groups, while the mRNA level of *bcl-xl* was markedly reduced in AOS0.05–0.15 groups. Furthermore, *caspase 9* mRNA level was markedly down-regulated in the AOS0.2 group. This result coincides with previous research on spotted sea bass, which pointed out that adding AOS to feed decreased mRNA of *caspase 3*, *caspase 8*, *caspase 9* and *bax*, and increased mRNA level of *bcl-2* [23]. In experiments involving zebrafish, the addition of AOS was found to reverse the expression of apoptosis-related genes induced by a high-fat diet (HFD), as demonstrated by significant up-regulation and down-regulation of *bcl-2* and *bax* mRNA levels, respectively [69]. The results show that by displaying the anti-apoptotic capability of AOS [19]. In addition, TUNEL staining and positive cell counting enabled the in situ observation of apoptotic cells and the microscopic quantitative analysis of apoptosis levels [70]. In this experiment, the apoptosis rate of intestinal cells was significantly lower in the AOS0.05–0.2 groups than in the control group. It is similar to the results of AOS reducing apoptosis in mouse cardiomyocytes [71]. The results of the TUNEL assay further confirm that AOS has a protective effect against excessive apoptosis in the intestine of juvenile largemouth bass. Therefore, adding AOS to the feed alleviate apoptosis in juvenile largemouth bass intestinal cells.

This study verified that dietary supplementation AOS exerted beneficial effects on growth, intestinal health, and anti-inflammatory and antioxidant status of largemouth bass. However, the present study still has several limitations; its regulatory mechanism was only preliminarily deduced from gene expression and histological data, restricted by experimental conditions, multi-omics analysis, protein quantification and pathway inhibition assays which were not included. Moreover, molecular samples were only collected at one timepoint, which limits the full illustration of dynamic regulatory pathways. Further research combining enzyme activity, protein abundance and metabolite profiles will help to comprehensively clarify the functional mechanism of AOS.

## 5. Conclusions

Proper dietary supplementation with AOS exerted positive effects on growth performance to a certain extent. According to SGR and WG second-degree polynomial regression analysis, the optimum AOS addition level for juvenile largemouth bass was 0.14–0.15%. In addition, proper dietary supplementation with AOS significantly improved FBW, WGR and SGR, enhanced intestinal digestive enzyme activities and antioxidant capacity, reduced intestinal MDA content and inflammatory response, inhibited intestinal cell apoptosis and DNA damage, and optimized intestinal morphological structure by increasing villus height, villus width and muscular thickness. However, several indicators were not significantly affected by AOS supplementation. No significant differences were observed in whole-body composition, FCR, SR and FI among groups. Additionally, plasma AST and ALT activities, intestinal anti-inflammatory factors (TGF-β, IL-10, IL-6), partial antioxidant indices (GPX, SOD, T-AOC), as well as related gene expressions (*C7A10A*, *C7A1A*, *C7A6*, *bax*, *il-8*, *tnf-α*, *fox*, *cat*, *zo-1*) remained unchanged. Taken together, proper dietary supplementation with AOS specifically improves the growth performance and intestinal health of largemouth bass to a certain extent, rather than exerting comprehensive regulatory effects on all physiological indicators. When considering practical production application, an AOS supplemental level of 0.14% is recommended.

## Figures and Tables

**Table 1:** Formulation and proximate composition of the experimental diets (% dry matter).

Ingredients	AOS0	AOS0.05	AOS0.1	AOS0.15	AOS0.2	AOS0.25
Fish meal	30	30	30	30	30	30
Chicken meal	5	5	5	5	5	5
Soy concentrated protein	8	8	8	8	8	8
Soybean meal	11	11	11	11	11	11
Rapeseed meal	7.2	7.2	7.2	7.2	7.2	7.2
Blood meal	5	5	5	5	5	5
Wheat meal	5	5	5	5	5	5
Corn gluten meal	7.7	7.7	7.7	7.7	7.7	7.7
Wheat gluten	3	3	3	3	3	3
Fish oil	6.66	6.66	6.66	6.66	6.66	6.66
Tapioca starch	5	5	5	5	5	5
Choline chloride	0.5	0.5	0.5	0.5	0.5	0.5
Vitamin premix	1	1	1	1	1	1
Mineral premix	1	1	1	1	1	1
Monocalcium phosphate	3.46	3.46	3.46	3.46	3.46	3.46
Vitamin C	0.05	0.05	0.05	0.05	0.05	0.05
L-Lysine	0.28	0.28	0.28	0.28	0.28	0.28
L-Methionine	0.15	0.15	0.15	0.15	0.15	0.15
AOS addition	0	0.05	0.1	0.15	0.2	0.25
Crude protein (%)	49.23 ± 0.49	49.23 ± 0.24	49.37 ± 0.28	49.94 ± 0.29	49.10 ± 0.89	48.82 ± 0.23
Crude lipid (%)	10.22 ± 1.48	10.16 ± 1.68	10.08 ± 1.86	10.49 ± 1.32	10.41 ± 1.64	10.03 ± 0.91
Gross energy (KJ/g)	19.87 ± 0.19	19.81 ± 0.23	19.69 ± 0.22	19.84 ± 0.19	19.75 ± 0.36	19.72 ± 0.28

Note: The crude protein contents of fish meal, chicken meal, soy concentrated protein, soybean meal, rapeseed meal, blood meal, corn gluten meal, and wheat gluten were 68.1%, 62.3%, 63.4%, 50.1%, 38.6%, 89.6%, 60.8% and 80.3%, respectively. These ingredients obtained from Wuxi Tongwei Feedstuffs Co., Ltd. (Wuxi, China). Vitamins and mineral premix (IU or mg/kg of premix), provided by Wuxi Tongwei Feedstuffs Co., Ltd. (Wuxi China). Vitamin premix (IU or mg/kg of premix): The contents of vitamin A, vitamin D_3_, vitamin E, vitamin K_3_, thiamin, riboflavin, calcium pantothenate, pyridoxine HCl, cyanocobalamin, biotin, folic acid, niacin, inositol, and vitamin C were 800,000 IU, 150,000–250,000 IU, 4500 IU, 600 mg, 800 mg, 800 mg, 2000 mg, 2500 mg, 8 mg, 16 mg, 400 mg, 2800 mg, 10,000 mg, and 10,000 mg. Mineral premix (g/kg of premix): The contents of calcium biphosphate, sodium chloride, potassium chloride, magnesium sulphate, ferrous sulphate, zinc sulphate, cupric sulphate, manganese sulphate, sodium selenate, cobalt chloride, and potassium iodide were 20 g, 2.6 g, 5 g, 2 g, 0.9 g, 0.06 g, 0.02, 0.03 g, 0.02 g, 0.05 g, and 0.004 g; zeolite was used as a carrier. Evonik Industries AG (Hanau, Germany) offered L-Methionine; Feeer Co., Ltd. (Shanghai, China) offered L-Lysine.

**Table 2:** Main methods and analysis equipment.

Indexes	Kits Model	Method	Testing Equipment/Assay Kits
Alkaline phosphatase (ALP)	/	International Federation of Clinical Chemistry recommended	Assay kits purchased from Mindray Medical International Ltd. (Shenzhen, China); Mindray BS-400 automatic biochemical analyzer (Mindray Medical International Ltd., Shenzhen, China).
Alanine transaminase (ALT) Aspartic transaminase (AST)			
Total protein levels Superoxide dismutase (SOD)	A045-2-2 A001-3-2	Bradford method WST-1 method	Assay kits purchased from Jiancheng Bioengineering Institute (Nanjing, China), Spectrophotometer (Thermo Fisher Multiskan GO, Shanghai, China).
Malondialdehyde (MDA)	A003-1-2	TBA method	
Catalase (CAT)	A007-1-1	Ammonium molybdenum acid method	
Total antioxidant capacity (T-AOC)	A015-2-1	ABTS method	
Glutathione peroxidase (GPX)	A005-1-2	Colorimetric method	
Amylase (AMS)	C016-1-1	Microplate method	
Lipase (LPS)	A054-1-1	Microplate method	
Trypsin (TRY)	A080-2-2	Ultraviolet colorimetric method	
Tumor necrosis factor-α (TNF-α)	H052-1-2	Double-antibody sandwich method	Assay kits purchased from Jiancheng Bioengineering Institute (Nanjing, China), Spectrophotometer (Thermo Fisher Multiskan GO, Shanghai, China).
Transforming growth factor β (TGF-β)	H034-1-2		
Interleukin 10 (IL-10)	ml832145		Assay kits purchased from Shanghai Enzyme-linked Biotechnology Co., Ltd. (Shanghai, China), Spectrophotometer (Thermo Fisher Multiskan GO, Shanghai, China).
Interleukin 6 (IL-6)	ml832141		

**Table 3:** Primer sequences for qRT-PCR analysis.

Gene	Forward Sequence (5′–3′)	Reverse Sequence (5′–3′)	Amplification Efficiency (%)	R^2^	Accession Number/Reference
*nf-* *κb*	CCACTCAGGTGTTGGAGCTT	TCCAGAGCACGACACACTTC	104.3	>0.99	XP_027136364.1
*tgf-β*	CACCAAGGAGATGCTGATT	CGTATGTTAGAGATGCTGAAG	105.1	>0.99	XM_038693206.1
*il-10*	CGGCACAGAAATCCCAGAGC	CAGCAGGCTCACAAAATAAACATCT	103.9	>0.99	XM_038696252.1
*il-8*	CGTTGAACAGACTGGGAGAGATG	AGTGGGATGGCTTCATTATCTTGT	104.6	>0.99	[33]
*tnf-α*	CTTCGTCTACAGCCAGGCATCG	TTTGGCACACCGACCTCACC	103.7	>0.99	XM_038710731.1
*keap1*	CGTACGTCCAGGCCTTACTC	TGACGGAAATAACCCCCTGC	101.1	>0.99	XP_018520553.1
*nrf2*	AGAGACATTCGCCGTAGA	TCGCAGTAGAGCAATCCT	101.3	>0.99	NM_212855.2
*gpx*	ATGGCTCTCATGACTGATCCAAA	GACCAACCAGGAACTTCTCAAA	102.8	>0.99	XM_038697220.1
*cat*	CTATGGCTCTCACACCTTC	TCCTCTACTGGCAGATTCT	108.4	>0.99	MK614708.1
*Mn-sod*	ACCATGCCACTTATGTCAACAAC	AAAGTCCCGCTTAATGGCCTC	102.4	>0.99	XM_038727054.1
*fox*	AGAGCTCCTGGTGGATGCTA	TAAGCAGGATACCCAGGGCT	99.0	>0.99	XM_038693765.1
*zo-1*	ATCTCAGCAGGGATTCGACG	CTTTTGCGGTGGCGTTGG	107.1	>0.99	XM_038701018.1
*occ*	GATATGGTGGCAGCTACGGT	TCCTACTGCGGACAGTGTTG	95.6	>0.99	XM_038715419.1
*clau*	CCAGGGAAGGGGAGCAATG	GСТCTТТGААССАGТGСGАС	100.0	>0.99	XM_038713307.1
*caspase 3*	GAGGCGATGGACAAGAGTCA	CACAGACGAATGAAGCGTGG	106.0	>0.99	XM_038713063.1
*caspase 8*	ACCAGGACCTGCTGTCATTG	TATCTGGAGATGCGCTGCTG	105.3	>0.99	XP_685430
*caspase 9*	CTGGAATGCCTTCAGGAGACGGG	GGGAGGGGCAAGACAACAGGGTG	109.2	>0.99	[34]
*bax*	ACTTTGGATTACCTGCGGGA	TGCCAGAAATCAGGAGCAGA	104.0	>0.99	[35]
*bxl-xl*	CAAGGAGGATGGGAACGCTT	TTCTGTGCAATGAGTCCCCC	99.0	>0.99	NP_571882
*bcl-2*	CCAACGTCATGGTTGTCATGG	GTGGAGCCAACCAGGAATCT	106.8	>0.99	Cluster-21914.31403
*pept1*	CCTATTTGCCTCGCTTTTGGTTGC	CATTAACCTTCGCCGTGAATTGGG	98.0	>0.99	MZ773078.1
*C7A5*	CGCTGCCGAACCCATTTTTG	TTGAGCGTGAGCGTCTTTGT	100.0	>0.99	XM_038706332.1
*C7A6*	TCCAGGTTGTTCTTCGTGGG	GCAGGGATCGGTGTGAATCT	103.7	>0.99	XM_038700945.1
*C7A1A*	GAGGAACCCGAAAGTGCTGA	CTCCAACAGCGTTGTGTGTG	106.7	>0.99	XM_038694119.1
*C7A10A*	CATTTGGCCCTTTTCCAGCC	CCTCAGCATGGCAGACAAGA	99.0	>0.99	XM_038701432.1
*C6A6*	TTTCAGTGCTTTCAGCCGGA	CCCACTTCAGCGGACCTATG	103.1	>0.99	MZ773077.1
*C7A8B*	CTTTGCTTACGGAGGCTGGA	GCGCGTGGTAGATTCCTGTA	107.4	>0.99	XM_038718738.1
*C6A14*	AGCTCATGGCTCTGATGTGTGT	TGGGATGCCCATGCAGAATA	102.3	>0.99	XM_038695490.1
*gapdh*	ACTGTCACTCCTCCATCTT	CACGGTTGCTGTATCCAA	106.2	>0.99	AZA04761.1

Note: *tgf-β*, transforming growth factor-β; *il-10*, interleukin-10; *il-8*, interleukin-8; *tnf-α*, tumor necrosis factor-α; *nf-κb*, nuclear factor kappa-β; *nrf2*, nuclear factor erythroid 2-related factor 2; *keap1*, recombinant kelch-like ech-associated protein 1; *gpx*, glutathione peroxidase; *cat*, catalase; *Mn-sod*, Mn superoxide dimutase; *clau*, claudin; *occ*, occludin; *zo-1*, zona occludens 1; *bax*, bcl2-associated x; *bxl-xl*, B-cell lymphoma-extra large; *bcl-2*, B-cell lymphoma-2; *pept1*, oligopeptide transporter 1; *C7A5*, L-type Amino Acid Transporter 1; *C7A6*, y+ type amino acid transporter 2; *C7A1A*, cationic amino acid transporter 1; *C7A10A*, amino acid transporter Asc-1; *C6A6*, BETA-amino acid transporter; *C7A8B*, L-amino acid transporter 2; *C6A14*, B^0,+^ amino acid transporter ATB (0, +); *gapdh*, glyceraldehyde-3-phosphate dehydrogenase.

**Table 4:** Growth performance of juvenile largemouth bass fed on alginate oligosaccharide (AOS)-supplemented diets for 56 days.

Parameters	AOS Supplementation Levels (%)						
	0	0.05	0.1	0.15	0.2	0.25	*p* Value
IBW (g)	9.38 ± 0.03	9.40 ± 0.09	9.45 ± 0.09	9.33 ± 0.06	9.37 ± 0.03	9.33 ± 0.06	0.257
FBW (g)	46.92 ± 2.50 ^a^	51.34 ± 3.60 ^ab^	53.18 ± 2.43 ^b^	53.54 ± 2.96 ^b^	52.15 ± 2.43 ^b^	49.02 ± 1.53 ^ab^	0.045
WGR (%)	289.49 ± 9.04 ^a^	317.54 ± 22.22 ^ab^	349.72 ± 32.9 ^ab^	380.23 ± 40.42 ^b^	398.34 ± 43.46 ^b^	320.29 ± 21.53 ^ab^	0.008
SGR (%/d)	2.55 ± 0.09 ^a^	2.69 ± 0.10 ^ab^	2.74 ± 0.08 ^b^	2.77 ± 0.10 ^b^	2.72 ± 0.08 ^b^	2.63 ± 0.05 ^ab^	0.041
FI (%/d)	2.20 ± 0.04	2.13 ± 0.10	2.19 ± 0.01	2.22 ± 0.17	2.09 ± 0.10	1.97 ± 0.12	0.117
FCR	1.11 ± 0.03	1.04 ± 0.04	1.03 ± 0.04	1.02 ± 0.11	1.00 ± 0.10	0.97 ± 0.05	0.060
SR (%)	90.00 ± 5.00	91.67 ± 7.64	93.33 ± 2.89	93.33 ± 7.64	91.67 ± 5.77	91.67 ± 5.77	0.982

Note: Data are presented as mean ± SD. Within each row, values bearing different superscript letters differ significantly (*p* < 0.05), whereas values sharing a common letter or without superscripts do not differ significantly (*p* > 0.05). Initial body weight (IBW, g), Final body weight (FBW, g), Weight gain rate (WGR, %) = 100 × (final weight (g) − initial weight (g))/initial weight (g), Specific growth rate (SGR, %/day) = 100 × ((Ln (final body weight (g)) − Ln (initial body weight (g)))/days), Feed conversion ratio (FCR) = dry feed fed (g)/(final body weight (g) − initial body weight (g)), Survival rate (SR, %) = 100 × (survival fish number at the end/total fish number at the start), Feed intake (FI, %/d) = 100 × dry feed fed (g)/[days × ((total final body weight (g) + total initial body weight (g))/2)].

**Table 5:** Whole-body composition (% on fresh weight basis) of juvenile largemouth bass fed on alginate oligosaccharide (AOS)-supplemented diets for 56 days.

Parameters	AOS Supplementation Levels (%)						
	0	0.05	0.1	0.15	0.2	0.25	*p* Value
Moisture (%)	72.47 ± 0.91	72.07 ± 0.56	72.40 ± 0.13	72.50 ± 0.25	71.87 ± 0.22	72.61 ± 0.50	0.480
Crude protein (%)	16.44 ± 0.36	16.75 ± 0.28	16.59 ± 0.19	16.30 ± 0.07	16.63 ± 0.10	16.14 ± 0.26	0.058
Crude lipid (%)	5.68 ± 0.53	6.10 ± 0.17	6.04 ± 0.67	5.85 ± 0.17	6.28 ± 0.74	5.52 ± 0.54	0.360
Crude ash (%)	3.47 ± 0.16	3.44 ± 0.06	3.53 ± 0.17	3.47 ± 0.10	3.56 ± 0.23	3.46 ± 0.10	0.921

Note: Data are presented as mean ± SD.

## Data Availability

The original contributions presented in this study are included in the article. Further inquiries can be directed to the corresponding authors.
